# Ameliorative Effect of Beraprost Sodium on Celecoxib Induced Cardiotoxicity in Rats

**Published:** 2018

**Authors:** Shafique Ahmad, Bibhu Prasad Panda, Mohammad Fahim, Neha Dhyani, Kiran Dubey

**Affiliations:** a *Department of Pharmacology, Faculty of Pharmacy, Jamia Hamdard, New Delhi, India. *; b *Department of Anatomy and Physiology, Hamdard Institute of Medical Sciences and Research, Jamia Hamdard, New Delhi, India.*; c *Department of Life Sciences, Indira Gandhi National Open University, New Delhi, India.*

**Keywords:** Beraprost Sodium, Cardiotoxicity, Celecoxib, Troponin-T, Tumor necrosis factor-α

## Abstract

Selective COX-2 inhibitors are most widely used analgesic and anti-inflammatory drugs; however, its maximal use is highly associated with various serious abnormal cardiovascular events. Beraprost sodium (BPS), prostacyclin analogue has been shown to vasodilatory, antiplatelates, anti-inflmmatory, and antioxidant activity. The objective of the present study was to evaluate the effect of BPS on celecoxib cardiotoxicity in rats. Toxicity was induced in male Albino rats (250-280 g) by celecoxib (100 mg/kg/day). BPS (30 μg/kg/day) was administered alone and in combination with celecoxib for 14 days and various biochemicals, hemodynamic, left ventricular, biochemical, and histopathological parameters were studied. Cardiotoxicity of celecoxib was revealed by a significant increase in serum lactate dehydrogenase (LDH), troponin-T (Tn-T), tumor necrosis factor-α (TNF- α), creatine kinase-MB (CK-MB) and systolic blood pressure (SBP), left ventricular end diastolic pressure (LVEDP), LV (dp/dt)_max_, and LV (dp/dt)_min_ as well as tissue thiobarbituric acid reactive substance (TBARS) and a significant decrease in tissue reduced glutathione (GSH). However, treatment with BPS reversed these alteration in LDH, Tn-T, TNF-α, CK-MB, SBP, LVEDP, LV (dp/dt)_max, _LV (dp/dt)_min, _TBARS and GSH levels. The histopathological study in cardiac left ventricle revealed protection of myocardium as manifested reduction of fibrosis by abolition of collagen deposition when celecoxib was combined with beraprost sodium. It could be concluded that beraprost sodium may prove a useful adjunct in patients being prescribed celecoxib.

## Introduction

Nonsteroidal anti-inflammatory drugs (NSAIDs) are most widely used therapeutic agents for the management of short and long term pain as well as in various inflammatory conditions. Unfortunately, its long term usage shows wide array of side effects including increased risk of cardiovascular complications (1). Inhibition of cyclo-oxygenase pathway plays an important role in the pain relieving mechanism as well as also in conversion of arachidonic acid into thromboxanes and prostaglandins (2). The coxibs (celecoxib) are potent cyclo-oxygenase-2 (COX-2) inhibitors that fall under the class of NSAIDs (3). Imbalance between the thromboxane A_2_ (TXA_2_) and prostacyclin (PGI_2_) levels is due to coxibs responsible for the thrombosis, atherosclerosis, and other cardiovascular abnormality (4). Evidences from the systematic review and meta-analysis suggested that use of celecoxib is associated with increased risk of myocardial infarction (5). Solomon *et al.* (6) in his randomized controlled study reported that long term use of celecoxib is linked with risk factor for the cardiovascular causes, heart failure, myocardial infarction, and stroke. Previously, it has been also claimed that effects on PGI_2 _and renal function due to selective COX-2 inhibitor with celecoxib administration might be associated with the abnormal blood pressure (7). Also, finding of Zaitone *et al.* (8) indicated that celecoxib could be responsible for the altered blood pressure which is an important hallmark of cardiovascular risk. 

Beraprost sodium (BPS) is a synthetic PGI_2_ receptor agonist and mimics similar to pharmacological profile as prostacyclin (9) such as vasodilatory effects (10), antiplatelates effects (11) , anti-thrombotic (12) , inhibition of vascular smooth muscle cells proliferation, and inflammatory cytokines production (13). Studies showed that beraprost sodium reduced the oxidative stress and inflammatory injury in diabetic cardiomyopathy model through p38 MAPK signalling pathway in high fat diet induced rats (14). BPS with its PGI_2_ receptor agonist potency inhibits TNF-α expression in lipopolysaccharide induced lung alveolar epithelial cells injury (15). In addition, Lefer *et al.* (16) reported that prostacyclin have cardio-protective effect during acute myocardial ischemia and reperfusion *in-vivo* and *in-vitro* animal paradigm.

However, effect of BPS and celecoxib against cardiovascular abnormalities has not been yet properly evaluated. Hence, the aim of present investigation was to evaluate the efficacy of BPS against celecoxib induced cardiovascular toxicity by assessing various indices such as hemodynamic, left ventricular function, Biochemical, and histological changes in experimental rats.

## Experimental

*Drugs and Chemicals*

Beraprost sodium was purchased from Sigma Aldrich, USA and Cayman Chemical, USA respectively. Celecoxib was procured as gift samples from Aurobindo Pharma Ltd. India. lactate-dehydrogenase (LDH) kit and creatine kinase (CK-MB) kit was purchased from Reckon Diagnostics Pvt. Ltd. ELISA Kits for estimations of Troponin-T (Tn-T) and Tumour necrosis factor-alpha (TNF-α) were obtained from Kinesis DX Ltd., USA and Krishgen biosystem Ltd., India respectively. All other chemicals used in the experiment were of analytical grade.

*Animals*

Adult male albino Wistar rats (250 to 280 g) were procured from the Central Animal House Facility, Hamdard University, New Delhi, India. The animals were kept in polypropylene cages under standard laboratory conditions (12-h night/dark cycles) and had free access to a commercial pellet diet and water *ad libitum*. The animal house temperature was maintained at 25 ± 2 °C. The protocol was approved by the Institutional Animal Ethics Committee and all studies were conducted under the guidelines of Committee for the Purpose of Control and Supervision of Experiments on Animals (CPCSEA), Government of India on animal experimentation (Protocol No-688/2010).

*Groups of animal*

The rats were divided into following groups (n = 8):

Group I: Control 0.5 mL/kg 1% CMC p.o. for 14 days.

Group-II: Celecoxib (CEL) *per se *100 mg/kg p.o. in 1% CMC for 14 days.

Group-III: Beraprost sodium (BPS)* per se *30 μg/kg p.o. for 14 days.

Group-VI: CEL+ BPS: Celecoxib 100 mg/kg p.o + Beraprost sodium 30 μg/kg p.o. for 14 days. 

Dose selection of celecoxib (100 mg/kg, p.o.) and beraprost sodium (30μg/kg, p.o) was based on the previous study (17, 18). After hemodynamic and left ventricular function measurement, the blood was withdrawn by cardiac puncture and the animals were sacrificed by cervical dislocation. The heart was rapidly removed and the separated serum from blood was stored at -80 °C for the assessment of various biochemical parameters. A heart of a rat in each group was removed and fixed in 10% formalin solution for the histopathology (Masson’s trichrome staining). 

*Hemodynamic and Left Ventricular Function Measurements*

At the end of treatment period, the animals were weighed and anaesthetized with urethane (1 g/kg, IP) Hemodynamic and left ventricular pressure was measured by using Powerlab Data Acquisition System (4/25, AD Instrument). The femoral artery was cannulated for measurement of systolic blood pressure (SBP), diastolic blood pressure (DBP), mean arterial pressure (MAP), and heart rate with a pressure transducer (Statham p23Db, CA, USA) and a micromanometer- tipped catheter (Millar Instruments) was placed in the left ventricle via right carotid artery to record left ventricular function; left ventricular systolic pressure (LVSP), left ventricular end diastolic pressure (LVEDP), LV (dp/dt) max and LV (dp/dt) min. Data were analysed by using acquisition data system (AD Instruments Pvt. Ltd. with software LABCHART 8 pro software, Australia (19).

**Table 1:** Hemodynamic parameters in control (Group I), CEL *per se *(Group II), BPS (Group III), CEL + BPS (Group IV).

**Group**	**SBP(mmHg)**	**DBP(mmHg)**	**MAP(mmHg)**	**HR (BPM)**
I	109.08 ± 2.35	83.22 ± 4.38	91.83 ± 3.53	322.06 ± 20.13
II	125.78 ± 4.00*	88.91 ± 2.61	101.19 ± 2.42	358.88 ± 16.58
III	102.45 ± 4.99	79.59 ± 2.56	88.73 ± 5.55	310.39 ± 9.76
IV	106.15 ± 2.56**	74.73 ± 2.80	86.37 ± 3.91	308.23 ± 18.81

Values are expressed as mean ± SEM (n = 8) and analyzed by one-way ANOVA followed by Tukey-Kramer multiple comparison test.

* *p* < 0.01 II v/s I and

** *p* < 0.01 IV v/s II. CEL: Celecoxib; BPS: Beraprost Sodium; SBP: systolic blood pressure; DBP: diastolic blood pressure; MAP: mean arterial pressure; HR: heart rate.

**Table 2:** Left ventricular function in control (Group I), CEL *per se *(Group II), BPS (Group III), CEL + BPS (Group IV).

**Group**	**LVSP (mmHg)**	**LVEDP (mmHg)**	**LV(dp/dt)**_max _ **(mmHgSec** ^-1^ **)**	**LV(dp/dt)**_min _ **(mmHgSec** ^-1^ **)**
I	109.10 ± 2.23	3.27 ± 0.44	10930.71 ± 824.12	-5892.81 ± 915.59
II	111.53 ± 1.96	10.21 ± 0.59**	6892.01 ± 407.67*	-3533.99 ± 314.10*
III	111.62 ± 3.07	6.28 ± 0.74	9970.62 ± 896.32	-5017.58 ± 497.15
IV	113.55 ± 1.54	4.49 ± 0.73*	11324.63 ± 875.04**	-6710.85 ± 688.41**

Values are expressed as mean ± SEM (n = 8) and analyzed by one-way ANOVA followed by Tukey-Kramer multiple comparison test.

* *p* < 0.05 II v/s I, IV v/s II and

** *p* < 0.01 II v/s I, IV v/s II . CEL: Celecoxib, BPS: Beraprost Sodium, LVSP: left ventricular systolic pressure, LVEDP: left ventricular end diastolic pressure, LV(dp/dt)_max_: maximum rate of rise of left ventricular function, LV(dp/dt)_min_: maximum rate of fall of left ventricular function.

**Table 3.** HW/BW and LVW/BW ratio in control (Group I), CEL *per se *(Group II), BPS (Group III), CEL + BPS (Group IV).

**Group**	**HW/BW**	**LVW/BW**
I	2.49 ± 0.11	1.10 ± 0.06
III	2.65 ± 0.13**	1.45 ± 0.049**
V	2.64 ± 0.06	1.01 ± 0.03
VI	2.60 ± 0.05*	1.11 ± 0.06**

Values are expressed as mean ± SEM (n = 8) and analyzed by one-way ANOVA followed by Tukey-Kramer multiple comparison test.

* *p* < 0.05 IV v/s II and

** *p* < 0.01 II v/s I, IV v/s II. CEL: Celecoxib, BPS: Beraprost Sodium, HW/BW: heart weight/body weight ratio, LVW/BW: left ventricle weight/body weight.

**Table 4:** Serum LDH and CK-MB in control (Group I), CEL *per se *(Group II), BPS (Group III), CEL + BPS (Group IV).

**Group**	**LDH (IU/L)**	**CK-MB (IU/L)**
I	78.30 ± 4.07	41.65 ± 2.87
II	107.47 ± 5.36*	66.17 ± 4.93**
III	74.16 ± 4.5	43.47 ± 2.90
IV	81.88 ± 4.32*	46.10 ± 2.77*

Values are expressed as mean ± SEM (n = 8) and analyzed by one-way ANOVA followed by Tukey-Kramer multiple comparison test.

* *p* < 0.05 II v/s I, IV v/s II and

** *p* < 0.01 II v/s I CEL: Celecoxib; BPS: Beraprost Sodium; LDH: lactate dehydrogenase; CK-MB: creatine kinase-MB.

**Figure 1:**
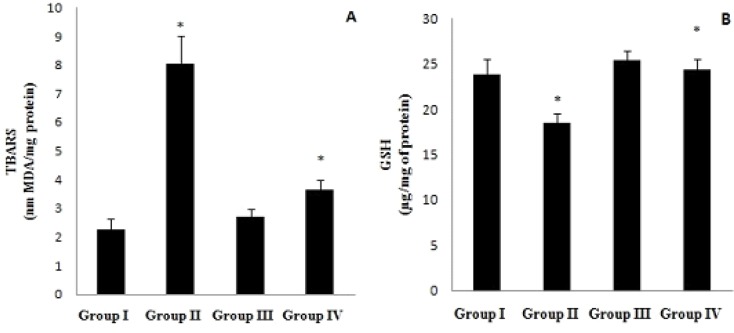
(A) Cardiac thio barbituric acid reactive substance (TBARS) level and (B) reduced glutathione (GSH) content activity in normal (Group I), CEL *per se *(Group II), BPS *per se* (Group III), and CEL + BPS (Group IV). Values are expressed as mean ± SEM (n = 8) and analyzed by one-way ANOVA followed by Tukey-Kramer multiple comparison test. *p < 0.05 II v/s I, IV v/s II. CEL: Celecoxib, BPS: Beraprost Sodium

**Figure 2:**
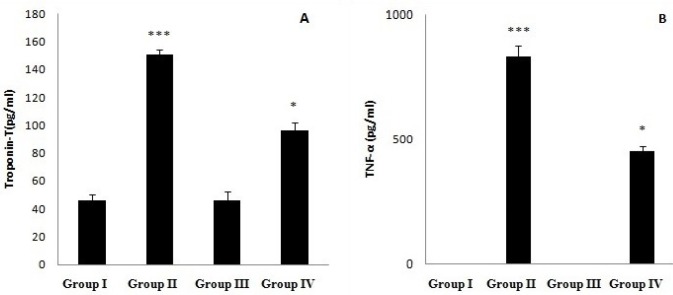
A) Serum Troponin-T (Tn-T) level and (B) Tumur Necrosis Factor-α (TNF- α) content activity in normal (Group I), CEL *per se *(Group II), BPS *per se* (Group III), CEL + BPS (Group IV)**. **Values are expressed as mean ± SEM (n = 8) and analyzed by one-way ANOVA followed by Tukey-Kramer multiple comparison test.*p < 0.05 IV v/s II, ***p < 0.001 II v/s I. CEL: Celecoxib, BPS: Beraprost Sodium

**Figure 3:**
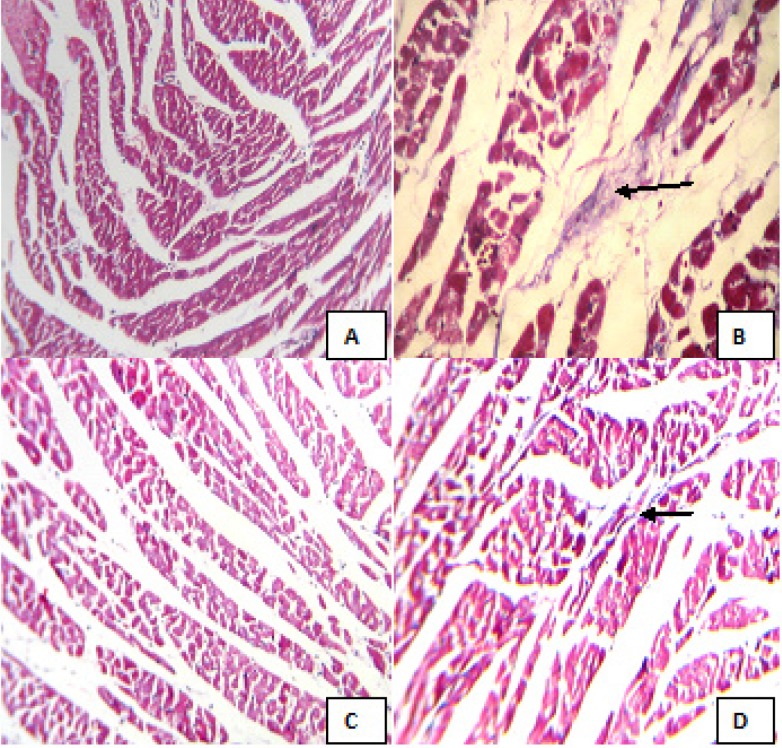
Masson’s trichrome stained sections of left ventricle in different animal groups. (A) Control (Group I), (B) CEL *per se *(Group II), (C) BPS *per se *(Group III), (D) CEL+ BPS (Group IV). (Masson’s trichrome stained; 100x); CEL: Celecoxib; BPS: Beraprost Sodium. Figures A and C show normal myocardium with no collagen. Figure B shows a focal area of collagen deposition. Figure D shows normal myocardium with very less collagen deposition

*Biochemical Estimations in Serum*

The serum LDH activity and CK-MB activity were determined by kinetic methods (20, 21) using respective kits and UV spectrophotometer (Model-150-200, Hitachi, Japan). Serum Tn-T and TNF-α was measured by using rat specific Tn-T (Kinesis DX Ltd. USA) and TNF-α (Krishgen biosystem Ltd. India) ELISA kits on ELISA reader (Micro Scan MS5608A, Electronic Corporation of India, Hyderabad).

*Biochemical Estimations in Cardiac Tissue*

All animals were sacrificed at the end of the study, *i.e.* on the 15^th^ day, the heart was immediately isolated. Tissue homogenates were prepared with 0.1 M tris-HCl buffer (pH 7.4) and supernatant of homogenates was employed to estimate reduced glutathione (GSH) (22), thiobarbituric acid reactive substance (TBARS) (23) and total protein (24) as described previously.

*Histopathological Evaluation*

The heart was fixed in 10% formalin solution for histopathological analysis. Formalin-fixed tissues (left ventricle) were embedded in paraffin, sectioned at 4 μm and stained with Masson’s trichrome. The sections were examined under light microscope, and the photomicrographs were taken. (25)

*Statistical analysis*

Statistical analysis was carried out using Graph Pad Prism 5.0 (Graph pad software; San Diego, CA). All data are expressed as mean ± standard error of mean (SEM). One-way analysis of variance (ANOVA) followed by Tukey-Kramer multiple comparisons test. *P* < 0.05 was considered as significant.

## Results

*Effect on Hemodynamic Parameters*

Treatment with CEL for 14 days significantly raised the SBP (*p* < 0.01) but not DBP, MAP and heart rate when compared with control. Furthermore, combination of BPS with CEL significantly (*p* < 0.01) reduced the SBP when compared with CEL (Table 1).

*Effect on left ventricular function*

Treatment with CEL also significantly increased (*p* < 0.01) the LVEDP and decreased (*p* < 0.05) the LV (dp/dt)_max_ and LV (dp/dt)_min _as compared to control. Whereas, combination of BPS with CEL significantly decreased (*p* < 0.05) LVEDP and increased (*p* < 0.01) the LV (dp/dt)_max _and LV (dp/dt)_min _when compared to CEL treatment alone group (Table 2).

*Effect on heart weight/body weight (HW/BW) and left ventricle weight/body weight (LVW/BW) ratio*

Treatment with CEL significantly raised the HW/BW (*p* < 0.05) and LVW/BW (*p* < 0.01) as compared to control. Whereas, the combination of BPS with CEL significantly decreased the HW/BW and LVW/BW (*p* < 0.05) when compared to CEL treatment alone (Table 3).

*Effect on LDH and CK-MB*

Treatment with CEL significantly raised the serum LDH (*p* < 0.05) and CK-MB (*p* < 0.01) as compared to control. Whereas, the combination of BPS with CEL significantly decreased (*p* < 0.05) the LDH and CK-MB level when compared to celecoxib treatment alone (Table 4).

*Effect on TBARS and GSH*

Treatment with CEL significantly raised the TBARS and GSH level (*p* < 0.01) as compared to control. Whereas, combination of BPS with CEL significantly (*p* < 0.05) decreased the TBARS and increased GSH level when compared to CEL treatment alone (Figure 1).

*Effect on Tn-T and TNF-α*

Treatment with CEL significantly (*p* < 0.001) raised the serum Tn-T and TNF-α level as compared to control. However, the combination of BPS with CEL significantly decreased (*p* < 0.05) the Troponin-T and TNF-α level when compared to celecoxib treatment alone (Figure 2).

*Effect on histopathology*

The masson’s trichrome staining of groups treated with 1% CMC (control) (A) and beraprost sodium (C) revealed normal myocardium with no collagen deposition. Whereas group treated with celecoxib (B) showed a focal area of collagen deposition. The combination of beraprost sodium with celecoxib (D) revealed normal myocardium with very less collagen deposition. (Figure 3).

## Discussion

Selective COX-2 inhibitors are widely used in the treatment of analgesic, anti-inflammatory, and antithrombotic (26), but its higher dose and long term use tightly associated with the myocardial infarction, atherosclerosis, heart failure and stroke (6). Thus, the present study was designed to investigate the effect of BPS on celecoxib induced cardiac toxicity in Wistar rats.

In our study, hemodynamic parameters were assessed for the better understanding of the celecoxib cardiotoxicity in cardiac biochemical and functional alterations. Selective COX-2 inhibitors, celecoxib showed slightly remarkable increased SBP (7). On the other hand, celecoxib failed to show any significant differences in heart rate, MAP, and DBP. In addition, it was also found that BPS treatment with celecoxib significantly demur the SBP level not heart rate, MAP and DBP when compared with the CEL alone group. 

Hypertension is the dire consequence of cardiovascular diseases and strongly allied with the left ventricle hypertrophy, a state in which cardiac ventricle walls become thick along with increased pressure overload induced cardiac abnormality (27). Furthermore, In the present study, celecoxib treatment shot up the LVEDP, decrease LV (dP/dT)_max_ and LV (dP/dT)_min_ which is an important tool for the diagnosis of cardiovascular diseases. Whereas, concurrent treatment of BPS with celecoxib significantly inhibit the increased LVEDP as compared to CEL treated groups.

In the present study, we found CEL treatment significantly increase the HW/BW and LVW/BW ratio, an important feature of cardiac hypertrophy development (28). Hypertrophy attributed to increased water content and oedema progression in intramuscular spaces, results in necrotic changes and inflammatory cells insult (29). However, it has been also found that BPS with celecoxib significantly reduced the HW/BW and LVW/BW.

Serum CK-MB and LDH is well known diagnostic enzymes marker of myocardial damage. Myocardial cells ruination due to insufficient oxygen supply or glucose, rupture cardiac membrane or make it permeable which leads to enzymes leakage and enters in to blood stream (30). In the current study, it was noticed that serum CK-MB and LDH elevation was observed in the COX-2 inhibitor, celecoxib treatment groups. This enhanced enzymes level in the serum advocate the myocardial membrane permeability deformities of celecoxib administration. Ueno *et al.* (18) reported that BPS defence of the myocardium against injury by suppressing enhanced CK-MB and LDH levels in occlusion/reperfusion injury animal model. Concurrent treatment of BPS with celecoxib attenuates the increased serum CK-MB and LDH.

GSH, a non enzymatic antioxidant is important in myocardial protection in response to free radical induced injury (28). Selective COX-2 inhibitors, celecoxib treated rats shows a significant change in the cardiac GSH level. However, treatment of BPS with celecoxib showed significant rise in cardiac GSH level. In line with our finding, Li *et al.* (14) also suggested that inhibition of ROS generation due to BPS could be associated with decreased GSH content in the cardiac tissue.

Lipid peroxidation involves oxidative deterioration of poly-unsaturated fatty acids associated with the abnormal membrane lipid bilayer arrangement and enzymes deactivation during myocardial ischemia (31). Increased TBARS is an important indicator of the ROS generation which might be associated with the oxidative tissue damage (32). These evidences were consistent with our findings. Elevated cardiac TBARS levels were also seen in celecoxib treated groups. BPS significantly decreased the cardiac TBARS levels in CEL that might be due to decreased lipid peroxides formation caused by oxidative stress which was supported by the previous studies (33).

Troponin T (Tn-T) considered as prognostic marker of drugs induced cardiac cell mishap in humans and animals (34). Enhanced troponin levels are important bacon for the increased coronary disease, suboptimal coronary flow, diminished left ventricular systolic function and assessment of infarct size in myocardial infarction (35). It has been reported that loss of membrane integrity due to cardiac myocytes injury could be the main reason for the cardiac troponin release in the blood stream (36). In our study, we found significant increase in serum Tn-T levels in CEL induced cardiotoxicity as compared to control rats which is in agreement with the previous findings (37). However, treatment of BPS with celecoxib shows the remarkably decreased Tn-T levels when compared with CEL groups.

TNF-α, a pro-inflammatory cytokines and chemokines serves a key function in apoptosis, necrosis, and cells proliferation (38). In the cardiac tissue TNF-α shows cardiotoxic effect and slows down the contractile function, decreased β- adrenergic inotropic response and promotes pro-apoptotic pathways (39). In addition, previous studies supported that oxidative stress is a key factor for the exacerbation of pro-inflammatory cytokines (40). In the present study, we observed that celecoxib enhance the serum TNF-α level in rats. Previous animal study also found that celecoxib treatment increases the TNF-α concentration in the alcohol liver steatosis (41). It is noteworthy that oral treatment of BPS along with celecoxib had a significantly lower serum TNF-α concentration compared to CEL induced cardiotoxicity groups. Several lines of studies revealed that BPS decrease TNF-α level via its anti-inflammatory and anti-apoptotic potency in myocardial tissue (14). Histopathological findings celecoxib shows a focal area of collagen deposition. However, administration of BPS with celecoxib significantly reduces collagen deposition.

In conclusion, the present study confirmed that celecoxib produced cardiotoxicity induced by in rats as evident by the release of myocyte injury markers in serum. Further studies are needed to clarify the precise pathway for the celecoxib induced cardiotoxicity. Moreover, the current study provided experimental suggestion that BPS maintained the antioxidant levels by inhibiting ROS generation and inflammatory cells and also it improved the myocardial events against high dose celecoxib. This investigation could be a scientific support to understand the better therapeutic approach of BPS on cardioprotection against myocardial injury. Therefore, BPS may be a suitable adjunct for those patients already with celecoxib prescription.
